# Emergence of Task-Related Motor Cortical Dysfunction in Mice with Progressive Parkinsonism

**DOI:** 10.64898/2026.05.29.728524

**Published:** 2026-06-02

**Authors:** Hiba Douja Chehade, Daniil Berezhnoi, Samhitha Somavarapu, Zihang Wang, Jitong Jiang, Liqiang Chen, Benjamin B. Risk, Thomas Wichmann, Hong-Yuan Chu

**Affiliations:** 1 Department of Pharmacology and Physiology, Georgetown University, Washington, D.C., United States; 2 Department of Biostatistics and Bioinformatics, Emory University, Atlanta, GA, United States; 3 Department of Neurology, School of Medicine, Emory University, Atlanta, GA, United States; 4 Emory National Primate Research Center, Emory University, Atlanta, GA, United States; 5 Aligning Science Across Parkinson’s (ASAP) Collaborative Research Network, Chevy Chase, MD, 20851

## Abstract

There are substantial functional changes in the primary motor cortex (M1) in Parkinson’s disease (PD). However, the temporal relationship between midbrain dopaminergic (DA) neurodegeneration, M1 circuit dysfunction, and Parkinsonian motor symptoms remains poorly understood. Using a genetic mouse model of progressive nigrostriatal DA degeneration (“MitoPark” mice), we determine the time course of M1 cellular dysfunction and skilled movement impairment as the midbrain DA neurons gradually degenerate. M1 pyramidal neuronal subtypes were identified using AAV-mediated retrograde labeling. During progressive DA loss, MitoPark mice developed gradually impaired performance in a reach-to-grasp single-food-pellet task. These impairments were detectable at a moderate motor stage of Parkinsonism. *In vivo* GCaMP6f imaging revealed that impaired skilled movement was associated with reduced cellular activity and movement responsiveness of M1 pyramidal neurons at a moderate motor stage of Parkinsonism. While both the corticospinal (CSp) and intratelencephalic (IT) neurons send glutamatergic inputs to the striatum, only the CSp neurons showed a selective and significant reduction in cellular activity and movement responsiveness during reaches. At the population level, we found that M1 pyramidal neurons include heterogeneous functional clusters with distinct temporal profiles in response to skilled movement. While movement encoding by different functional clusters is longitudinally stable in control mice, it degrades and diverges significantly in MitoPark mice. The impaired stability is further supported by a longitudinal analysis of individual neuronal activity related to movements. Together, these results provide novel insights into the emergence of M1 circuit pathophysiology at cellular and neural population levels during progressive Parkinsonism.

## Introduction

Parkinson’s disease (PD) is a chronically progressive neurological disorder associated with a reduced number and amplitude of spontaneous movements. These hypokinetic motor signs are causally linked with the degeneration of dopamine (DA) neurons in the substantia nigra pars compacta (SNc) and the accompanying DA depletion throughout the basal ganglia ^1,2^. According to the traditional model of the pathophysiology of PD, the loss of DA neuromodulation has opposing effects on the activity of direct and indirect pathways of the basal ganglia, resulting in abnormally increased basal ganglia GABAergic inhibition of their downstream targets, including the thalamocortical network and the brainstem ^1–4^. In addition, the loss of DA neuromodulation induces complex anatomical and functional adaptations at the cellular and synaptic levels in the striatum ^5–7^, the globus pallidus ^8,9^, and the subthalamic nucleus ^8,10–12^. Overall, these changes promote the emergence of aberrantly synchronous burst patterns of neural activity throughout the basal ganglia and the thalamocortical network ^1,2,6,13^. Compelling evidence suggests that the suppression of abnormally synchronized network activity is correlated with the therapeutic effects of deep-brain electrical stimulation ^2,14^, supporting its pathological role in the pathophysiology of PD.

Patients with PD not only exhibit overall slowness of movement (i.e., bradykinesia) but also deficits in fine motor skills (e.g., micrographia) ^15^. Interestingly, in PD patients, impairment in manual dexterity likely occurs independently of other clinical measures of disease severity ^16^, indicating that the mechanisms underlying impairment in motor skills and general motor activity may not entirely overlap. It is known that the acquisition and execution of fine motor skills are highly dependent on cerebral cortical function ^16,17^. Relevant to the pathophysiology of PD, the primary motor cortex (M1) and the supplementary motor area and premotor cortices are major sources of synaptic input to the basal ganglia, and they are also the primary downstream targets of basal ganglia GABAergic inhibition via the thalamus. In the Parkinsonian state, M1 exhibits anatomical and functional alterations ^18–25^. Functional imaging studies have reported altered cortical activity and abnormal cortical plasticity in PD patients ^25,26^. Animal model studies have provided additional mechanistic insight into cortical dysfunction following DA loss, including reduced firing rate of subsets of cortical neurons ^18,22^, enhanced synchronous firing ^21–23,27^, and abnormal movement encoding ^23,28^.

Most prior preclinical studies on cortical circuit dysfunction were conducted using subacute or chronic neurotoxin models of fully developed Parkinsonism. These models do not adequately recapitulate the chronic, progressive nature of PD or exhibit robust intersubject variability in disease staging, making it impossible to capture stage-dependent changes in cortical circuits. Therefore, it is necessary to define the temporal relationship between dopaminergic degeneration, changes in cortical activity, and abnormalities in skilled movements in a chronically progressive model of the disease to better understand cortical dysfunction in PD.

In the present work, we used a progressive mouse model of nigrostriatal degeneration (i.e., MitoPark mice ^29,30^) to conduct longitudinal cellular imaging of neural activity in the M1 in freely moving animals during gradually worsening striatal DA depletion. Our results demonstrate that, during the progression of Parkinsonism, M1 neurons gradually start to show abnormal activity at both cellular and population levels, with the corticospinal (CSp) neurons showing a more robust and selective disruption in their activity than the intratelencephalic (IT) neurons. In studies of task-related activity, we also demonstrated that, as a whole population, M1 pyramidal neurons comprise distinct functional clusters for movement encoding, whose organization is disrupted as Parkinsonism develops in MitoPark mice. Together, the present work provides new insight into cell-subtype-specific adaptations in M1 circuits during the progressive loss of midbrain dopaminergic neurons in the parkinsonian state.

## Results

**Figure 1.**
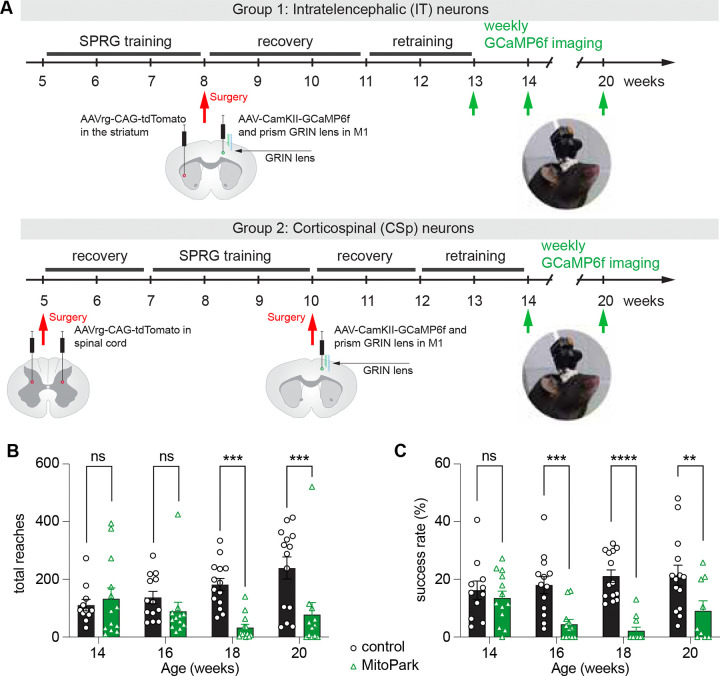
Skilled movement deteriorates during progressive loss of midbrain DA neurons. A) Diagram showing the overall experimental design. B-C) Performance of MitoPark mice and controls in the SPRG task. Total number of reaches (B) and success rate (C) significantly decrease in MitoPark mice compared to littermate controls. Linear mixed models with fixed effects for group, age (a factor with four levels), and the group-by-age interaction, with a random intercept for individual mouse, were used to compare MitoPark versus control across weeks. Within each model, the Benjamini-Hochberg correction was applied across the age-specific group comparisons. Results are reported as mean ± SEM hereafter. Significance codes: * p < 0.05, ** p < 0.01, *** p < 0.001, **** p < 0.0001. Detailed numbers and statistics are available in the Source Data Table.

**Figure 2.**
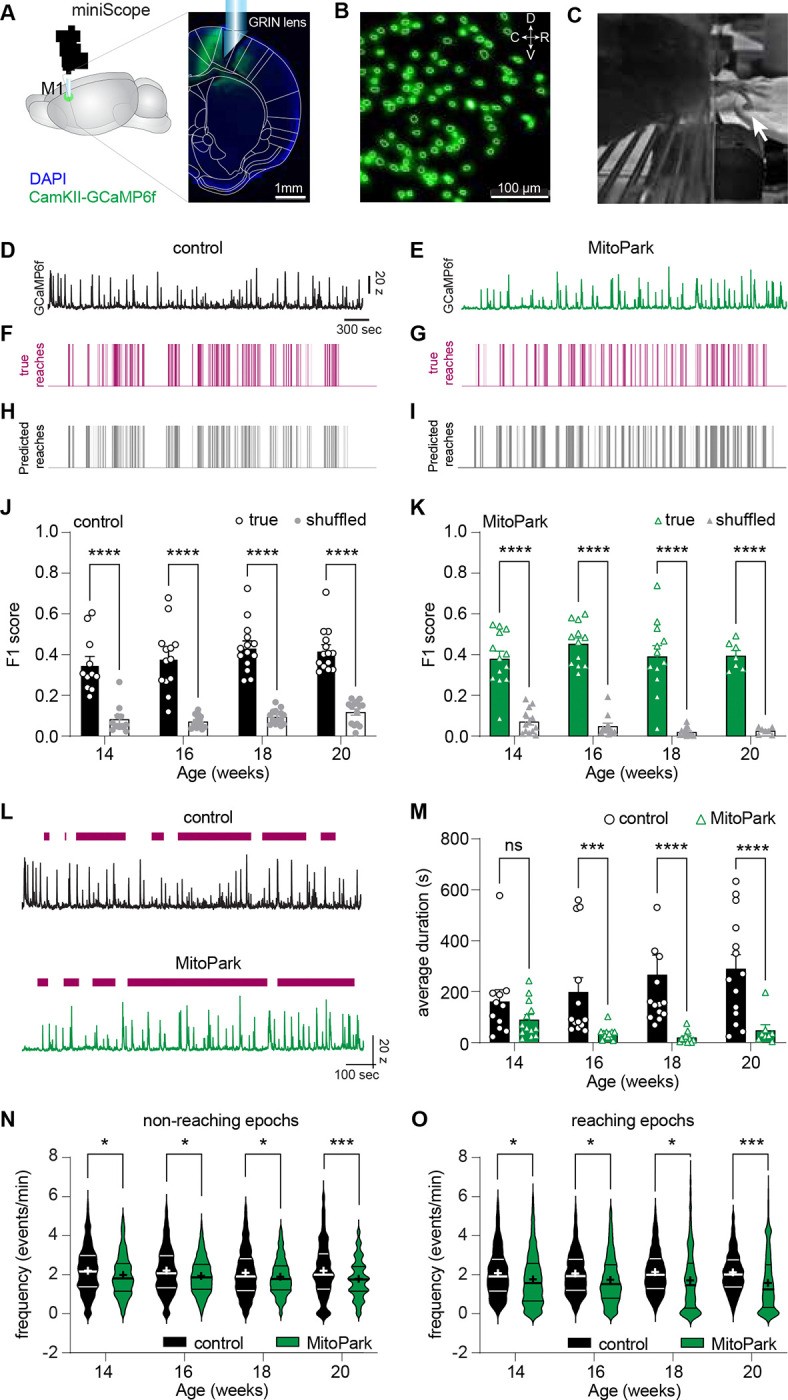
Decreased cortical pyramidal neuronal activity during progressive Parkinsonism. **A)** Diagram showing *in vivo* Ca^2+^ imaging from M1 and a representative histological image showing GCaMP6f expression and lens placement in M1. **B**) Maximal projection image of Ca^2+^ transients from a segmented field of view of the Prism GRIN lens. **C**) An example behavior video frame while a mouse was performing reach-to-grasp task, highlighting the grasp onset (white arrow). **D-G**) Extracted GCaMP6f traces of a representative neuron from control (D) and MitoPark (E) mice, and the corresponding time sampled reaches (F-G). **H-I**) Predicted reaches by the RUSBoost algorithm using the recorded GCaMP6f signals in D, E. **J-K**) M1 pyramidal neuron population F1 score is greater for reaches than for shuffled behavior data for both control (J) and MitoPark (K) mice across ages. Linear mixed-effect models with fixed effects of task (reaching or shuffle), age, and their interaction, with random intercepts for mouse and age nested within mouse to account for repeated and paired measurements. **L**) Representative GCaMP6f traces from control and MitoPark mice. Horizontal lines on top indicate the reaching epochs. **M**) MitoPark mice exhibited a gradually decreased duration of reaching epochs relative to littermates. Linear mixed-effects model with log-transformed duration and fixed effects for group, age, and their interaction, with a random intercept for mouse. **N-O**) Frequency of Ca^2+^ events from M1 pyramidal neurons during non-reaching (N) and reaching (O) epochs is reduced in MitoPark mice compared to littermate controls. Horizontal lines indicate median and interquartile range (IQR), and cross (+) symbols indicate the mean. For each behavioral state, linear mixed-effects model with fixed effects for group, age, and their interaction, with random intercepts for mouse and for age nested within mouse to account for repeated measurements. Within each model, the Benjamini-Hochberg correction was applied across the age-specific group comparisons. In N and O, the group-by-age interaction was not significant, and the p-value from the group effect in the model with no interaction is reported. Detailed numbers and statistics are available in the Source Data Table.

We used MitoPark mice as a model of progressive nigrostriatal dopaminergic neurodegeneration to longitudinally investigate M1 circuit dysfunction and its relationship to Parkinsonian motor deficits ^30^. We trained MitoPark mice and littermates to acquire and execute a self-initiated single-pellet reaching-and-grasping (SPRG) task (Fig. 1A, Key Resource Table)^31^, which is recognized as a cortex-dependent skilled movement in rodents^17,32,33^. Following the successful acquisition of the task, mice were subjected to *in vivo* GCaMP6f imaging to monitor M1 pyramidal neurons during the SPRG task (20 min/session/week, Fig. 1A) using a miniature endoscope (Inscopix, Fig. 2A). Given the well-characterized time course of neurodegeneration and behavioral changes in MitoPark mice ^29,30,34^, we selected 14-, 16-, 18-, and 20-week-old animals to define the temporal relationship between striatal DA depletion and cortical activity changes and its potential correlation with the development of skilled movement deficits at different stages of Parkinsonism.

### Skilled movement deteriorates during progressive loss of midbrain DA neurons

We trained MitoPark mice and littermate controls to learn the SPRG task beginning at 5 or 7 weeks of age (depending on the experimental groups, Fig. 1A) prior to stereotaxic surgery to implant a gradient refractive index (GRIN) lens in M1 at 8–10 weeks of age. Once recovered from surgery, MitoPark mice showed performance similar to that of littermate controls on the task at 14 weeks of age (Fig. 1B–C), indicating their intact capacity to acquire the task. Starting at as early as 16 weeks of age, MitoPark mice showed significantly impaired performance relative to littermate controls, and this impairment worsened over time (Fig. 1B, C). Specifically, both the total number of reaches and the proportion of successful reaches decreased in MitoPark mice relative to littermate controls (Fig. 1B, C). Given the well-established time course of general motor deficits in MitoPark mice, the above data suggest that loss of midbrain DA neurons impairs the generation of skilled movements, occurring after the onset of bradykinesia-like symptoms at 12–14 weeks of age ^29,30,34^.

### Decreased cortical neuronal activity during progressive Parkinsonism

Layer 5 (L5) pyramidal neurons connect the cortex with the striatum and subcortical motor regions, playing a critical role in the execution of movement ^35–39^. We conducted a longitudinal analysis of M1 neuronal activity using *in vivo* GCaMP6f imaging. To this end, we injected AAV9-CaMKII-GCaMP6f into the caudal forelimb region of the M1, followed by a subsequent implantation of a GRIN lens at the same location of AAV injections (Fig. 2A, B). *Post*mortem histological analyses showed that (1) GCaMP6f expressed robustly in the forelimb region of M1^40^ (Supplementary Fig. 1A, B); (2) the implantation consistently centered at the L5 of M1, corresponding to location of the retrogradely labelled CSp neurons via spinal injection of AAVrg-tdTomato (Fig. 2A, Supplementary Fig. 1A–C), (3) there were no differences in the rostrocaudal, mediolateral, or dorsolateral coordinates of the bottom edges of the GRIN lenses in control and MitoPark mice (Supplementary Fig. 1D–F); and (4) MitoPark mice showed a substantial reduction in the striatal tyrosine-hydroxylase (TH) positive axon terminals at the end of recording (21–24 weeks of age), confirming striatal dopamine loss (Supplementary Fig. 1G–I).

GCaMP6f signals were recorded and extracted from M1 pyramidal neurons in 14-, 16-, 18-, and 20-week-old MitoPark mice and littermates. During the continuous GCaMP6f recording, the animals moved freely in the reaching box. We sought to determine whether skilled movements are encoded by GCaMP6f signals in M1 pyramidal neurons and whether this encoding is disrupted by progressive DA loss. To do this, we implemented a supervised machine learning model, the RUSBoost classifier ^41^, to decode reaching behavior using simultaneously recorded M1 neuronal population activity (Fig. 2D–I). Using the F1 score as a measure of overall performance of the classification model (see Materials and Methods), we found that M1 population activity better predicted reaches than shuffled behavioral data in both littermates and MitoPark mice of different ages (Fig. 2J, K). The above data suggest that, as in the healthy state, pyramidal neuronal activity in M1 critically contributes to the internally-generated skilled movements in MitoPark mice across stages of Parkinsonism.

Next, we quantified the rate and amplitude of Ca^2+^ events as indirect measures of neuronal activity and compared them across behavioral states between MitoPark mice and littermate controls. First, we identified “non-reaching epochs” in the recorded videos, defined as periods lasting at least 30 sec during which animals did not perform reaches. Remaining epochs during which animals performed active reaches were then defined as active “reaching epochs” (Fig. 2L). The duration of reach epochs declined gradually as SNc DA neurons progressively degenerated in MitoPark mice, compared with littermate controls (Fig. 2M), consistent with the reduction of the total number of reaches from early to late stages of MitoPark mice (Fig. 1B).

During non-reach epochs, animals were in behavioral states presumably less dependent on the motor cortex ^37,42^. We found a significant group effect in the frequency of Ca^2+^ events (p = 0.0003). Specifically, the frequency of spontaneous Ca^2+^ events decreased by 12% at 14 weeks, 11% at 16 weeks, 10% at 18 weeks, and 20% at 20 weeks in MitoPark mice compared with age-matched littermate controls (Fig. 2N). Similarly, there was a significant group effect during the reach epochs (p = 0.0003), which was associated with a 15% reduction in Ca^2+^ events at 14 weeks, 16% at 16 weeks, 20% at 18 weeks, and 25% at 20 weeks (Fig. 2O). We also quantified the area under the curve (AUC) of the identified Ca^2+^ events as an integrated measure of neuronal activity. We found no difference in the AUC of Ca^2+^ events during either non-reach or reach epochs in MitoPark mice relative to littermate controls (Supplementary Fig. 2B–C). Together, these data demonstrate that the activity of M1 pyramidal neurons, as a population, decreased gradually and significantly from the early to the advanced motor stages in Parkinsonism, and that these changes were more pronounced when skilled movements were conducted.

### Cell-subtype-specific decreases in neuronal activity in Parkinsonism

**Figure 3.**
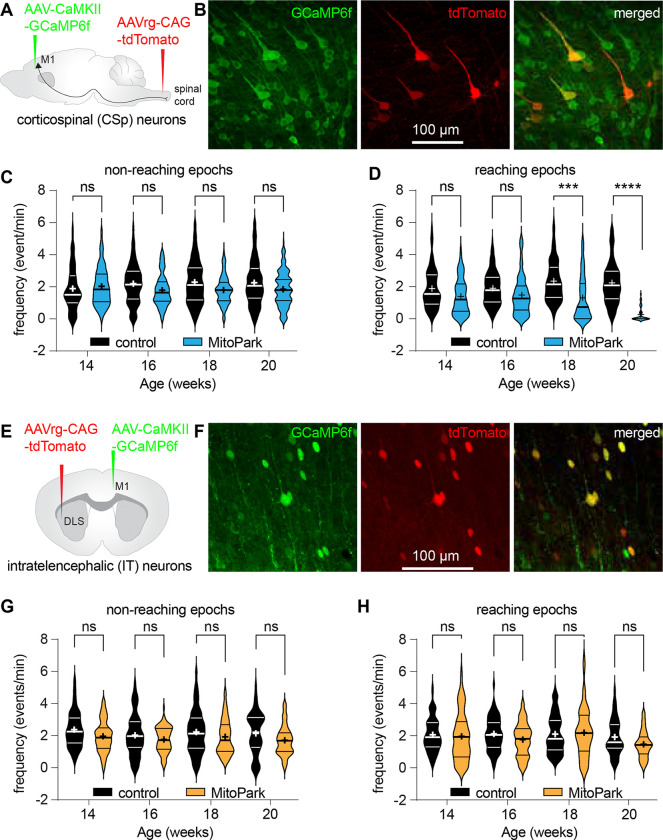
Cell-subtype-specific decreases in neuronal activity in Parkinsonism. **A)** Strategy to express GCaMP6f in all pyramidal neurons in M1 and retrogradely label CSp neurons. **B)** Representative images showing AAV expression in M1. Middle panel shows CSp neurons labeled with tdTomato in M1 ipsilateral to the injection site in the spinal cord. **C-D)** Frequency of Ca^2+^ events from M1 CSp neurons is not significantly changed during non-reaching epochs (C), but is progressively reduced in reaching epochs (D), in MitoPark mice compared to littermate controls. **E)** Strategy to express GCaMP6f in all pyramidal neurons in M1 and retrogradely label IT neurons. **F)** Representative images showing AAV expression in M1. Middle panel shows IT neurons labeled with tdTomato in M1 contralateral to the injection site in the DLS. **G-H)** Frequency of Ca^2+^ events from M1 IT neurons during non-reaching (G) and reaching (H) epochs. Horizontal lines indicate median and IQR, cross (+) symbols indicate the mean. For the non-reaching task (C and G), a linear mixed-effects model with fixed effects for cell subtype, age, group, their pairwise interactions, and their three-way interaction, with random intercepts for mouse and for age nested within mouse to account for repeated measurements was fit. For the reaching task (D and H), the same mixed-effects structure was used. Within each model, the Benjamini-Hochberg correction was applied across the age-specific group comparisons. Detailed numbers and statistics are available in the Source Data Table.

Considering the cellular heterogeneity of cortical pyramidal neurons, we next examined whether subtypes of M1 pyramidal neurons are differentially affected by loss of DA, as reported previously ^18,22,23^. Specifically, we focused on the CSp and IT neurons in layer 5 that connect M1 with the basal ganglia circuits (Fig. 3A, E). CSp and IT neurons were retrogradely labeled by injection AAVrg-CAG-tdTomato into the spinal cord and contralateral striatum, respectively. *In vivo* dual-color imaging of GCaMP6f and tdTomato signals allowed us to monitor tdTomato-labeled CSp or IT neurons (Fig. 3B, F) by quantifying their activity changes during the development of Parkinsonism.

We analyzed Ca^2+^ events of both neuronal subtypes during the non-reach and reach epochs separately. In non-reach epochs, the frequency of Ca^2+^ events in CSp or IT neurons was comparable between control and MitoPark mice across ages (Fig. 3C, G). In contrast, while the animals were actively reaching, CSp neurons exhibited an age-dependent reduction in Ca^2+^ event frequency in MitoPark mice relative to littermate controls (Fig. 3D), but no such alteration was observed in the IT neurons between groups (Fig. 3H). Specifically, CSp neurons showed comparable frequencies of Ca^2+^ events between 14-week-old MitoPark mice and littermate controls. CSp neurons from MitoPark mice showed a progressive decline in the frequency of Ca^2+^ events, with reductions of 22%, 45%, and 88% at 16-, 18, and 20-weeks of age, respectively, relative to littermate controls (Fig. 3D). In both reach and non-reach epochs, there were no consistent or robust changes in the AUC of Ca^2+^ events in either CSp or IT neurons from MitoPark mice relative to littermate controls (Supplementary Fig. 2D–G).

We conclude that the activity of CSp neurons decreased gradually in MitoPark mice, correlating with the onset of impaired motor skills as Parkinsonism developed. The hypoactivity of M1 CSp neurons, but not IT neurons, is partially consistent with predictions from the traditional model and prior electrophysiological studies in non-human primates (NHPs) ^22,23^, further highlighting cell-subtype-specific alterations in M1 in Parkinsonism. Because the overall activity of IT neurons did not change in MitoPark mice, they were not included in further analyses in the present study.

### Unaltered functional connectivity between CSp neurons in Parkinsonism

Prior electrophysiological studies suggested that cortical pyramidal neurons appear to be abnormally synchronized in Parkinsonism ^21^. To follow up on this issue, we assessed the functional connectivity between the CSp and IT neurons (separately) in control and MitoPark mice. Neural synchrony between cells was quantified using the Pearson correlation coefficient computed from the extracted raw Ca^2+^ traces from both reach and non-reach epochs (See Materials and Methods). Surprisingly, we found no group differences in the distribution of Pearson correlation coefficients, neither for CSp (Supplementary Fig.3 A, B) nor IT neurons (Supplementary Fig.3 C, D) during either reach or non-reach epochs across ages. Together, the above data suggest that the synchrony of Ca^2+^ events between M1 pyramidal neurons is not altered in MitoPark mice relative to controls, though this is likely due to the insufficient temporal resolution of GCaM6f imaging.

### Impaired movement encoding by CSp neurons in Parkinsonism

**Figure 4.**
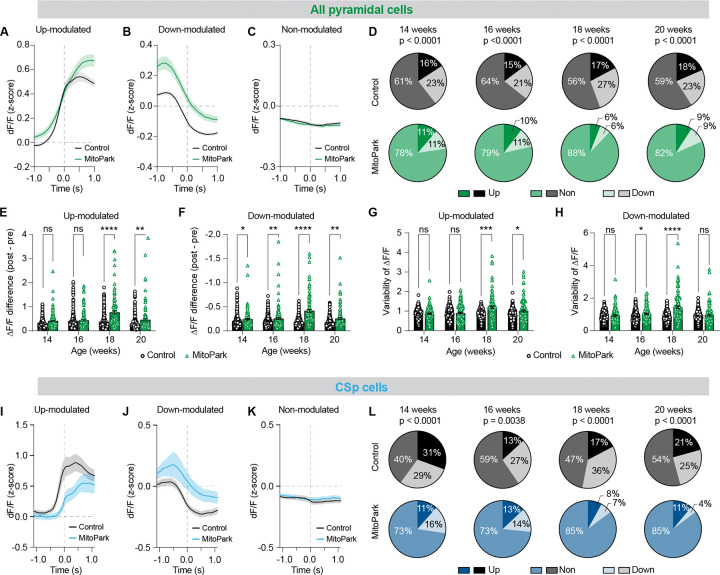
Impaired modulation of pyramidal neurons by movement in Parkinsonism. **A-C)** Representative mean calcium activity of M1 pyramidal neurons in MitoPark and control mice at 16 weeks of age, showing up- (A), down- (B), and non-modulated (C) categories. Gray dashed vertical lines indicate the grasp onset. **D)** Proportions of different categories of pyramidal neurons at each time point. **E-F)** Summary results of the magnitude of modulation for up-modulated (E) and down-modulated (F) pyramidal neurons. **G-H)** Summary results of the variability of ΔF/F (standard deviation across trials) averaged before grasp onset for up-modulated (G) and down-modulated (H) pyramidal cells. **I-K)** Representative mean Ca^2+^ activity of CSp neurons in MitoPark and control mice at 16 weeks of age, showing up- (I), down- (J), and non-modulated (K) categories. Gray dashed vertical lines indicate the grasp onsets. **L)** Proportions of different categories of CSp neurons at each time point. Chi-square test to compare differences in D and L. In E-H, linear mixed-effects models for log-transformed data were used to compare group differences with fixed effects for group, age, and their interaction, and random intercepts for mouse and age nested within mouse to account for repeated measurements.

The data above demonstrate that M1 pyramidal neurons, particularly CSp neurons, showed greater activity changes during the reach epoch than during the non-reach epoch (Fig. 2C, D), suggesting a possible impairment in their control of skilled movements. To study the relationship between the activity of all GCaMP6f-expressing M1 pyramidal neurons (regardless of whether they were CSp or IT neurons) and the generation of reaches, we performed peri-movement analyses by aligning *z*-scored Ca^2+^ signals (see Materials and Methods) to the onset of the grasps, which could be reliably identified from the behavior videos (Fig. 2C). For the peri-movement analysis, we statistically compared GCaMP6f signals within a time window of ±1.0 s at the onset of grasps. Based on the direction of GCaMP6f changes, M1 neurons were classified into up- or down-modulated groups (Fig. 4A–B) corresponding to those with statistically increased or decreased amplitude of Ca^2+^ signals within the ±1 sec time window. M1 neurons that showed unaltered magnitudes of Ca^2+^ signals at the onset of grasp were classified as the non-modulated group (Fig. 4C)^36^. 56%–64% of M1 pyramidal neurons in control mice across ages were characterized as non-modulated. This proportion exceeded 75% in 14- and 16-week-old MitoPark mice and > 80% in the 18- and 20-week-old groups (Fig. 4D). These data demonstrate that the proportion of movement-modulated cortical pyramidal neurons decreases gradually during progressive Parkinsonism. To quantify the magnitude of movement-related modulation in pyramidal neurons, the difference in GCaMP6f fluorescence was calculated between pre- and post-grasp onset for both up- and down-modulated groups. In the up-modulated group, we found greater changes in the magnitude of GCaMP6f signals in MitoPark mice compared to littermate controls at 18 and 20 weeks of age (Fig. 4E). Similarly, in the down-modulated group, we found greater changes in the magnitude of GCaMP6f signals in MitoPark mice relative to littermate controls across ages (Fig. 4F), indicating an early alteration in this subgroup of neurons.

In healthy animals, the generation of skilled reaching movements is associated with coordinated cortical neuronal activity, likely driven by synaptic inputs ^32,33,43–45^. We examined the variability of Ca^2+^ signals across all recorded pyramidal neurons during the task as an indirect measure of population-level neuronal synchronization. While mice performed internally-initiated reaches in the freely moving paradigm, they initially oriented their heads and snouts toward the food slit and adjusted their whole bodies and postures to be ready to reach and grasp^46^. This stereotyped preparatory period represents a relatively consistent and conservative behavioral state. The GCaMP6f signal within the 1 second immediately preceding grasp onset was used to quantify the variability of neuronal activity (i.e., the standard deviation of ΔF/F across trials). In the up-modulated neurons (Fig. 4G), we observed a greater variability in MitoPark mice than in controls, which was statistically significant at 18 and 20 weeks of age. In the down-modulated neurons (Fig. 4H), a greater variability of neuronal activity in MitoPark mice than in controls was detected at 16 and 18 weeks of age. These observations indicate that cortical pyramidal neurons may have become less synchronized at the onset of grasps, perhaps because synaptic inputs are less effective at driving and synchronizing cortical pyramidal neuronal activity after DA loss^19^.

Given the selective alterations in CSp neurons (Fig. 3), we further characterized their activity changes during reaching in MitoPark mice (Fig. 4I–L). CSp neurons could also be categorized into up-, down-, or non-modulated groups (Fig. 4I–K). In control animals, ~50% of CSp neurons were classified as non-modulated (Fig. 4L). This result is consistent with earlier reports showing that CSp exhibit a heterogeneous relationship between their activity and movement ^36^. As Parkinsonism developed, the proportion of non-modulated CSp neurons gradually increased in MitoPark mice from the early to late stages, and this increase was significantly higher than in littermate controls (Fig. 4L).

We quantified the amplitude of modulation and variability of neuronal activity in up- and down-modulated CSp neurons. While their GCaMP6f signal changes showed a trend of increased magnitude of modulation in MitoPark mice relative to controls, such differences did not reach statistical significance (Supplementary Fig. 4A). Similarly, we did not find robust alterations in the variability of CSp neuronal activity in either up- or down-modulated CSp neurons from MitoPark mice relative to those from littermate controls across ages (Supplementary Fig. 4C–D). Notably, the progressive Parkinsonism in MitoPark mice led to a significant decline in the number of reaches, rendering the dataset underpowered for robust analysis. Together, the above results suggest that M1 pyramidal neurons, particularly the CSp neurons, showed decreased movement engagement and increased population-level response variability during the progressive loss of SN DA neurons, perhaps indicative of impaired movement-related activity in the Parkinsonian state.

### Longitudinal stability of movement encoding by individual neurons in M1.

**Figure 5.**
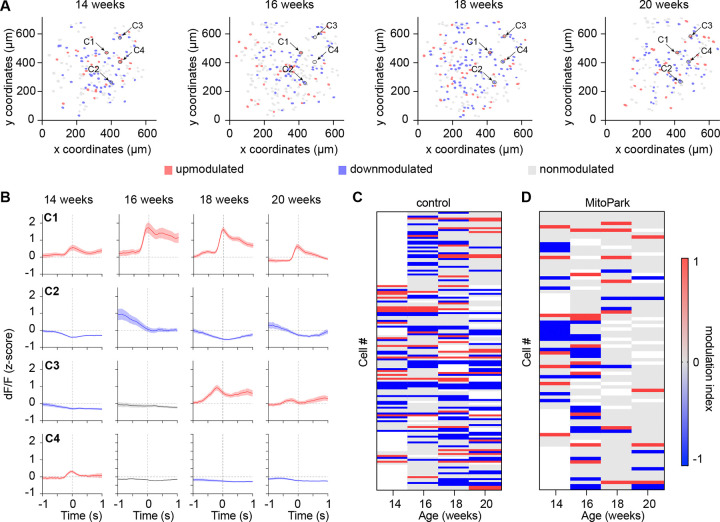
Longitudinal stability of movement encoding by individual CSp neurons in M1. **A-B)** Representative cell maps (A) and modulation traces (B) from one control mouse, with color coding indicating up-, down-, and non-modulated cells, in red, blue, and gray, respectively. Circled cells in A are the longitudinally registered CSp cells represented in B, which shows the mean modulation across trials at different timepoints. C1 and C2 are respectively up- and down-modulated across time (stable), whereas C3 and C4 modulation changes across time (unstable). Gray dashed vertical lines indicate the grasp onset. **C-D)** Heatmaps showing modulation of CSp cells registered in at least 3 timepoints, with the same color coding as A and B, for control (C) and MitoPark (D) mice.

Leveraging the spatial cellular resolution and stability of miniature scope technology, we followed a subset of CSp neurons from 14 to 20 weeks of age in both control and MitoPark mice, allowing us to study movement-encoding by their activity over time (Fig. 5A). Although the relationship between CSp activity and movement is dynamic and can be altered over time, as reported previously ^36,38,45^, a small number of CSp neurons maintained their movement modulation categories over time (Fig. 5A, B), perhaps representing an ensemble of cells important for the analyzed movements ^43^. In control mice, 4% and 10% of up- and down-modulated CSp neurons, respectively, maintained their movement-modulation categories across ≥3 age groups between 14 and 20 weeks (Fig. 5B, C). In contrast, none of the up- or down-modulated CSp neurons could be repeatedly categorized into the same movement modulation category across≥ 3 ages between 14 and 20 weeks in MitoPark mice (Fig. 5D). These data indicate that the response stability of movement-related activities of individual CSp neurons was gradually impaired in MitoPark mice relative to controls. Quantitatively, we calculated the Fleiss Kappa to assess the stability of movement-modulation by CSp neurons. We found a Fleiss Kappa of 0.27 for CSp neurons in the control group between 14 and 20 weeks, i.e., weak but detectable temporal stability. In MitoPark mice, the Fleiss kappa for CSp neurons was 0.1 between 14 and 20 weeks, indicating no reliable temporal stability.

### Population encoding of movement by M1 neurons is impaired in Parkinsonism

**Figure 6.**
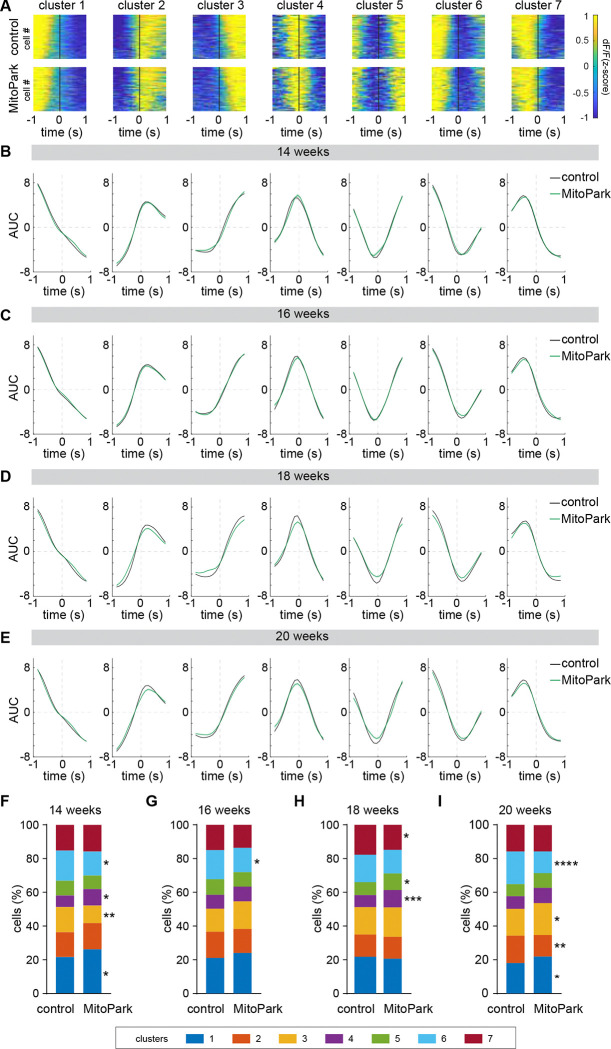
Altered movement encoding by functional clusters of M1 pyramidal neurons in MitoPark mice relative to controls. **A)** Heatmaps of ΔF/F of the different functional clusters of M1 pyramidal cells, aligned to the grasp onset (black vertical line), in control (top) and MitoPark (bottom) mice. **B-E**) Area under the curve (AUC) of ΔF/F calculated within ±1 sec aligned to grasp onset (gray vertical line). (**F-I**) Cell proportions per cluster in control vs MitoPark mice across ages. The Chi-square test was used to compare groups. Detailed numbers and statistics are available in the Source Data Table.

Given the decreased proportion of M1 neurons responsive to movement and their instability in encoding the reach event over time, we sought to understand how these changes affect the dynamic control of skilled movement at the population level. We characterized the temporal dynamics of the denoised Ca^2+^ signals from all GCaMP6f-expressing cortical pyramidal neurons during reaches using an unsupervised spectral clustering algorithm ^47,48^. Based on their distinct temporal dynamics, cortical pyramidal neurons can be categorized into 7 functional subpopulations (referred to as clusters hereinafter) in both control and MitoPark mice (Fig. 6A–E). These clusters were well separated in a high-dimensional principal component space (Supplementary Fig. 5A–C) and displayed peak activity at different timings during reaches (Supplementary Fig.5D). This analysis suggests the existence of functionally distinct clusters of M1 pyramidal neurons in relationship to the generation of skilled movements. Interestingly, while clusters 2 and 3 overlapped largely with the activity-based up-modulated category, clusters 1, 6, and 7 overlapped with the down-modulated category. All clusters partially overlapped with the non-modulated category (Supplementary Fig. 5E). These data suggest that the activity-based categories of cortical neurons (Fig. 4) comprise diverse functional clusters based on their temporal response profiles during reaches (Supplementary Fig. 5E).

These data support the notion that distinguishable dynamic temporal profiles of movement-related activity of pyramidal neurons exist. Interactions between these subpopulations of M1 pyramidal neurons may contribute to the generation of reaching activity. Thus, we quantified the proportions of cells in each cluster and found significant differences between MitoPark mice and littermate controls across ages (Fig. 6F–I). In particular, the proportion of pyramidal neurons across different clusters remained relatively consistent in control mice of different ages. In contrast, MitoPark mice exhibited longitudinal fluctuations in these proportions between 14 and 20 weeks, resulting in significant group differences in 4 of 7 clusters at 20 weeks (Fig. 6F–I). These results suggest that as Parkinsonism develops, movement-related activity patterns across functional clusters of M1 pyramidal neurons are strongly altered, in line with the reduced overall stability of movement encoding in the activity-based categories of neurons in MitoPark mice (Fig. 5).

**Figure 7.**
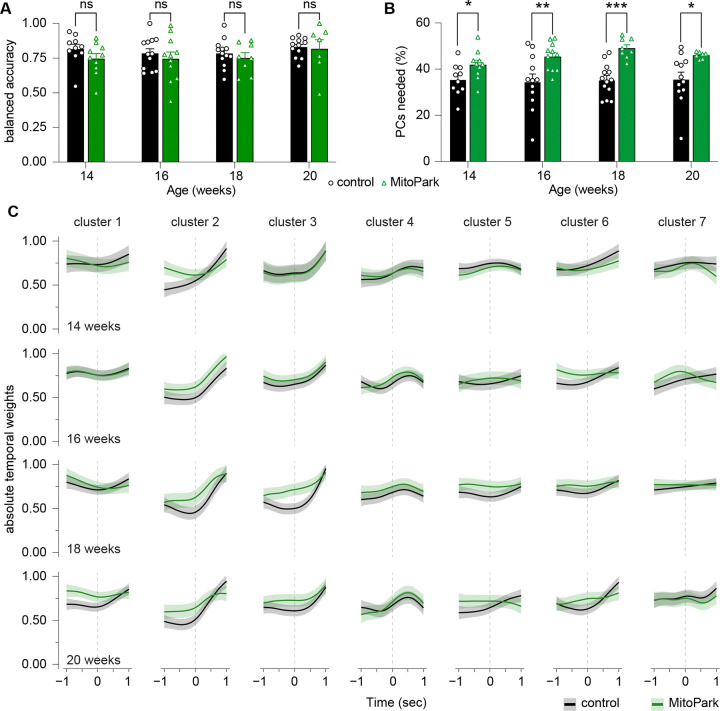
Predictive modeling indicates an abnormal cortical neuronal encoding of movement in progressive Parkinsonism. **A)** No difference was observed between MitoPark and controls in the balanced accuracy of the binomial logistic regression model, averaged across folds. **B)** The proportion of PCs needed to explain 85% of the variance in PCA is significantly higher in MitoPark mice relative to controls. (A-B) Linear mixed-effects model, followed by Benjamini-Hochberg correction. **C)** Absolute temporal weights of the different clusters from the logistic regression model in control and MitoPark mice across ages. GAMM with autocorrelated errors was fitted for each functional cluster at each age, with separate smooths for time in MitoPark and control. Shown are the fitted smooth curves and point-wise 95% confidence bands. The difference curves are shown in the supplementary Fig. 7. Detailed numbers and statistics are available in the Source Data Table.

Next, we implemented binomial logistic regression to assess the contributions of the different clusters to reach decoding. We ran the regression analysis on time-resolved Ca^2+^ data from reach epochs and randomly sampled non-reach epochs with matched duration (See Methods). This model reliably distinguished reach from non-reach segments across mice and ages (Fig. 7A), as indicated by consistently high balanced accuracy (a metric that evaluates how well the model identifies reach/non-reach segments). Classifier performance was further evaluated by examining the variability of model weights across folds, which was minimal, and by inspecting confusion matrices, which consistently showed high fractions of correct predictions (Supplementary Fig. 6). Together, the above results are consistent with the results from the RUSboost classifier (Fig. 2), suggesting an overall critical role of M1 pyramidal neurons in the performance of skilled movements in both control and MitoPark mice. Interestingly, although balanced accuracy in MitoPark mice was comparable to that of controls even as they aged (Fig. 7A), M1 pyramidal neuron activity in MitoPark mice required a significantly larger proportion of principal components to capture 85% of the variance compared with controls (Fig.7B). These data suggest that neuronal population activity in MitoPark mice is less dominated by a small number of coordinated activity patterns, with variance distributed more diffusely across principal components.

Furthermore, we explored possible changes in the contributions of different clusters to movement decoding by comparing the absolute model weights across ages (as a measure of each cluster's contribution to the model's outcome) in function of time around grasp onset (Fig. 7C). We found divergence in clusters’ contribution to movement decoding between MitoPark mice and littermate controls, particularly at 18 and 20 weeks of age (Fig. 7C, supplementary Fig. 7). Together, these data demonstrate the impaired stability of movement-related activity of M1 pyramidal neurons at both individual neuron and populational levels in progressive Parkinsonism.

## Discussion

In the present work, we studied neuronal activity changes in M1 and examined their relationship to impairment of skilled movement during the progression of Parkinsonism. We found that skilled motor activity declines gradually in MitoPark mice, associated with decreased activity of M1 pyramidal neurons. At the cell-subtype level, M1 CSp neuronal activity is preferentially reduced over time in MitoPark mice. In contrast, the activity of IT neurons remains intact. These observations support the conclusion that M1 circuits develop cell subtype-specific alterations in Parkinsonism ^18,19,22,23^. We found that ~50% of CSp neurons are modulated by skilled movement in healthy controls, and that this proportion decreases gradually as Parkinsonism progresses in MitoPark mice. Longitudinally, we found that CSp neuron activity in relation to movement becomes more dynamic and less stable as Parkinsonism develops in MitoPark mice. At the population level, M1 pyramidal neurons can be categorized into distinct functional clusters based on their temporal profiles relative to movements. Moreover, the dynamic contribution of these clusters to movement decoding appears to differ gradually and significantly between MitoPark mice and controls. Together, this work consolidates the concept that M1 exhibits cell-subtype- and circuit-specific adaptations that likely contribute to impairments in skilled movements following chronic, progressive striatal DA depletion. Furthermore, the present study reveals an abnormal and unstable relationship between M1 neuronal activity and the generation of movement under Parkinsonian states ^21^.

### Cell-subtype-specific M1 circuit changes in Parkinsonism

Although the traditional circuit model of PD pathophysiology predicts that M1 is hypoactive due to abnormal basal ganglia inhibition following midbrain dopamine neuron loss, emerging evidence suggests that M1 circuits develop cell-subtype- and input-specific adaptations in Parkinsonism ^18,19,22,23^. Using MitoPark mice as a model of progressive Parkinsonism, we provide additional information on the time course of M1 circuit changes in freely moving animals. We confirm that striatal DA depletion selectively reduces CSp neuronal activity *in vivo* but does not affect IT neuronal activity. Thus, both *in vivo* and *ex vivo* electrophysiological and Ca^2+^ imaging studies demonstrate that the parkinsonism-related neuronal dysfunction in M1 differs by cell type ^18,19,22^. In addition, we found that CSp neurons are hypoactive in 16-week-old MitoPark mice with nearly complete striatal DA depletion and moderate impairment of general motor function. These observations suggest that early inventions that restore DA function in the basal ganglia or cortex (e.g., dopaminergic medications) may ameliorate cortical circuit changes and preserve cortex-dependent function in PD.

### Altered movement modulation by CSp neurons in Parkinsonism

CSp neurons directly connect the M1 circuit with subcortical motor regions and critically contribute to the execution of movement ^36,49^, but they are relatively less studied using optical imaging approaches compared to layer 2/3 IT neurons ^37,43,50^. This is at least partially due to the deep location of CSp neurons, making them less accessible with *in vivo* two-photon imaging. By implanting a prism GRIN lens, we were able to monitor deep CSp neuronal activity in freely moving control and Parkinsonian mice for months. In control mice, we found significant heterogeneity in the relationship between the activity of CSp neurons and movement. Specifically, the activities of ~50% of CSp neurons showed no correlation with the monitored skilled movement, whereas the activities of the remaining proportion of CSp neurons correlate positively or negatively with movement. Such heterogeneity in the correlation between CSp activity and movement has also been reported in prior research ^36,51^. It is noteworthy that the activity of a large proportion of CSp neurons is modulated by well-learned skilled movements in control mice, indicating their role in motor execution. This is distinct from the gradual disengagement of L2/3 pyramidal neurons from learned movement over time ^50^. During progressive Parkinsonism, the proportion of movement-modulated cortical pyramidal cells, particularly CSp neurons, gradually decreased. In addition, these neurons also showed a less stable temporal relationship to movements in MitoPark mice than in controls. Furthermore, we found that the proportions of cortical pyramidal neurons within distinct functional clusters (Fig. 6) were significantly altered in MitoPark mice compared with littermate controls. Together with the longitudinal tracking of a subset of cortical pyramidal neurons (Fig. 5), these data suggest a reduced stability of the movement-related cortical neuronal activity during progressive parkinsonism. Combined with the impaired spine dynamics of M1 pyramidal neurons and changes in their synaptic input ^19,28,33,43,52^, as well as other functional impairments ^32,38,46,53–55^, these M1 microcircuit adaptations during the progression of Parkinsonism may be important determinants of the cardinal motor signs of PD.

### Cellular and circuit mechanisms of subtype-specific dysfunction in M1

In healthy controls, population-level coordination of M1 neurons results in the emergence of functional ensembles that underlie skilled movement performance, a process involving synaptic plasticity ^43^. Striatal DA depletion triggers cellular and synaptic adaptations throughout the basal ganglia nuclei and the thalamocortical network. Specifically, loss of midbrain DA neurons leads to reduced thalamic inputs to M1 and downregulation of the intrinsic excitability of M1 pyramidal tract neurons in Parkinsonian NHPs and rodents^18,19,24^. Under physiological conditions, the firing of cortical pyramidal neurons is primarily driven by synchronous thalamic synaptic inputs, which are critical for the execution of skilled movement ^32,33,56^. In Parkinsonian conditions, weakened thalamic excitatory inputs are likely to lead to a breakdown of the thalamocortical network, resulting in less coordinated and more independent M1 neuronal activity during movement. If this is the case, it could partially explain the reduced proportion of M1 pyramidal neurons responsive to skilled movement (Fig. 4D, L), the increased variability in their responses (Fig. 4G, H), and the higher proportion of principal components needed to explain 85% of the variance (Fig. 7) in MitoPark mice relative to controls.

Additionally, the altered movement encoding by functional clusters of M1 pyramidal neurons likely involves intricate interactions among synaptic excitation and inhibition as well as cellular excitability ^51,57^. In the present study, animals underwent extended training to perform uncued skilled movements. It has been reported that inactivation of premotor regions impairs the performance of such internally-generated skilled movement ^55^. Our unpublished observation indicates that cortical input from the secondary motor cortex to M1 decreases significantly following DA loss. Thus, while the worsened motor performance is accompanied by decreased pyramidal neuronal activity, other impairments, such as changes in corticocortical interactions, may contribute to Parkinsonian motor signs.

### Limitation of the present work

While longitudinal *in vivo* recordings from freely moving mice are a technical strength of the present work^58^, freely moving mice initiate and execute internally generated reach-to-grasp behavior at a self-defined pace, making data analysis challenging. One limitation of the present work is the reduced number of reaches as Parkinsonism progresses in MitoPark mice, making it impossible to study the relationship between M1 activity and different reach categories (e.g., successful v.s. failed) due to the small sample size, particularly in animals with advanced Parkinsonism. Another limitation of the Ca^2+^ imaging approach is its low temporal resolution, which renders detailed studies of the relationship between individual neuronal activity patterns and movement parameters, spike-by-spike assessments of inter-neuronal synchrony, and tests of high-frequency oscillatory neuronal activity impossible. However, as shown in this study, Ca^2+^ imaging provides valuable information that advances our understanding of PD pathophysiology, especially in longitudinal studies of defined groups of neurons, which may help guide the design of effective cortex-based therapies for the disease.

## Material and Methods

All animal studies were reviewed and approved by the Georgetown University Institutional Animal Care and Use Committee (IACUC protocol #2024–0001).

### Animals.

DAT^Cre^ mice (JAX stock #006660) were crossed with Tfam^fl/fl^ mice (JAX stock #026123) to obtain double heterozygous mice (DAT^Cre/+^, Tfam^fl/+^), which were then crossed with homozygous Tfam^fl/fl^ mice to obtain MitoPark mice (DAT^cre/+^, Tfam^fl/fl^) for experiments ^29,30^. Mice were housed up to 5 animals/cage under a 12h/12h light/dark cycle with free access to food and water in accordance with NIH guidelines for animal care and use.

### Stereotaxic surgery.

For *in vivo* cell-subtype visualization, we performed stereotaxic surgery at 8 or 10 weeks of age to retrogradely label subtypes of M1 pyramidal neurons based on their long-range axonal projections. Specifically, mice were placed on a stereotaxic frame (RWD, Model: 71000) under 2–3% isoflurane for anesthesia and were subcutaneously injected with carprofen (5 mg/kg) or buprenorphine (3.25 mg/kg) for analgesia. Body temperature was maintained and monitored using a heating pad connected to a feedback controller. To record calcium activity from cortical excitatory neurons, AAV9-CaMKII-GCaMP6f (Addgene #100834-AAV9, RRID: Addgene_100834-AAV9; volume = 300 nl, titer = 1 × 10^13^ GC/ml) was injected in M1 contralateral to the dominant paw [from bregma in mm, anteroposterior (AP) = +0.3 and +0.8, mediolateral (ML) = 1.25, dorsoventral (DV) = −1.0 from dura]. To retrogradely label IT neurons, AAVrg-CAG-tdtomato (Addgene #59462-AAVrg, RRID: Addgene_59462-AAVrg; volume = 500 nl, titer = 7 × 10^12^ GC/ml) was injected in the dorsolateral striatum (DLS) that was contralateral to the M1 injections (in mm, AP = +0.7, ML = 2.3, DV = −2.6, and AP = +1.0, ML = 2.0, DV = −2.6 from dura, 500 nl per injection). Details of intracranial injection surgery can be found on Protocols.io (dx.doi.org/10.17504/protocols.io.rm7vzye28lx1/v1). To retrogradely label corticospinal neurons in M1, AAVrg-CAG-tdtomato (Addgene #59462-AAVrg, RRID: Addgene_59462-AAVrg; titer = 7 × 10^12^ GC/ml) was injected in spinal C7–8 segments (ML = ±0.5 from midline and DV = −0.7 from dura). Details of spinal cord surgery can be found on Protocol.io: dx.doi.org/10.17504/protocols.io.81wgbz5e3gpk/v1. Mice used for IT neuron studies were trained to perform the SPRG task between 5 and 8 weeks of age (see below), followed by AAV injections (Fig. 1A). Considering the potential spinal cord damage associated with invasive surgery that may impair the acquisition of motor skills, mice for CSp neuron studies were subject to AAV injections into the spinal cord at 5 weeks of age, and then allowed to recover for 2 weeks before motor training between 7 and 10 weeks of age (Fig. 1A). Once mice acquired the SPRG skills, they received AAV9-CaMKII-GCaMP6f injections and Prism lenses implantations in M1 (Fig. 1A). After the surgery, all mice received an additional 1–2 weeks of retraining to ensure that they could perform the SPRG task.

### GRIN lens implantation.

Mice were mounted on a stereotaxic frame (RWD, Model: 71000) under 2–3% isoflurane anesthesia and subcutaneously injected with carprofen (5 mg/kg) or buprenorphine (3.25 mg/kg) for analgesia. Body temperature was maintained and monitored using a heating pad connected to a feedback controller. A prism GRIN lens attached to a baseplate (Cat # 1050–004419, Inscopix) was inserted in M1 at the AAV injection site centered at (in mm) AP = +0.5, ML = 1.0, DV = −0.5, so that the imaging plane would be parallel to the midline and faces M1 medially. The lens was held using a dummy microscope (Inscopix, Cat # 1050–003762) and lowered at 2 μm/min. The lens and baseplate were fixed to the skull using C&B Metabond dental cement (Parkell, Farmingdale, NY), and a baseplate cover (Inscopix, Cat # 1050–004639) was placed to protect the lens. Mice with lens implantation were single-housed after surgery. Details of lens implantation surgery can be found here: https://www.protocols.io/view/injection-of-aav-and-prism-grin-lens-implantation-4r3l21rrqg1y/v1.

### Behavior training and data analysis.

Details of the construction of the behavior training box and the SPRG task training protocol were described in our methods paper ^31^. Briefly, mice were habituated to the experimenter and the customized training box for 5 days. Then, mice were food-restricted to keep the body weight at 80% of baseline until the end of training. Mice underwent 2 to 3 days of shaping sessions (20 min), during which they learned to use their forepaws rather than their tongues to obtain pellets. The dominant forepaw was identified as the one used for more than 70% of the reaches. Mice that consistently grasped pellets using their paws entered the training phase. This included daily 20-minute sessions over 7 days, during which the mice were trained to use only their dominant paws to grasp the pellets. Forepaw movement was recorded using a high-speed FLIR camera (Teledyne FLIR, Model: Blackfly S) that was triggered by the custom task controller when the animal approached the frontal slit and placed its paws on a metal bar. Behavioral videos were reviewed by an experimenter blinded to the animal genotypes using BORIS software (Behavioral Observation Research Interactive Software, University of Torino, RRID:SCR_025700) to manually identify overall reaches and successful reaches, as well as to timestamp the onset of grasp (i.e., the peak timing of reaches) for peri-event reaching analysis. Details of the behavior training protocol can be found here: https://www.protocols.io/view/mouse-reach-to-grab-task-training-protocol-ewov11bb2vr2/v1. To compare behavioral variables between MitoPark mice and controls across weeks (Fig. 1B, C), a linear mixed-effects model with fixed effects for group, age (a factor with four levels), and the group-by-age interaction, with a random intercept for mouse to account for repeated measures was fitted. We then used Kenward-Roger degrees of freedom and Benjamini-Hochberg correction was applied across the age-specific group comparisons.

### *In vivo* Ca^2+^ imaging.

*In vivo* calcium imaging was performed using nVue 1.0 (Inscopix, Cat # 1000–005735) and nVue 2.0 (Inscopix, Cat # 1000–006810) systems. Two weeks post-surgery, the mice were placed in the behavior box daily for 7 days to re-habituate to the setup with a miniscope attached to the baseplate. At the end of the habituation, the mice were fasted overnight, followed by *in vivo* imaging the next day. During the imaging session, mice were placed in the behavior box with the miniscope attached to the baseplate. While mice were in the training box, the GCaMP6f signal was recorded continuously at 20 Hz using the Inscopix Data Acquisition System by setting LED power at 0.2 −0.4 mW/mm^2^, exposure time at 50 ms, and sensor gain of 2 to 4. During recording, transistor–transistor logic (TTL) pulses (5 V) were generated by the behavioral camera and sent to the nVue system to synchronize GCaMP6f recordings and behavior videos. Digital focus is manually chosen to obtain more cells in the field of view, typically between 0 and 1000 Inscopix units (i.e., ~ 0–300 μm). The static red channel videos were also recorded for 5 sec at multiple focal planes for cell-subtype identification. At the end of the recording session, the mice were returned to their cages with food *ad libitum*. Details of Ca^2+^ imaging protocol can be found here: dx.doi.org/10.17504/protocols.io.yxmvm1w5nv3p/v1

### Ca^2+^ data analysis.

All image processing was performed with Inscopix Data Processing Software (IDPS). Spatial downsampling by a factor of 2 was applied to the images for efficient data processing, whereas no temporal downsampling was applied. Videos were motion-corrected, and cell traces were extracted using the CNMF-E algorithm ^59^, which is widely used for background-fluctuation correction of one-photon calcium imaging data. Cell traces and contours extracted by CNMF-E were manually checked and artefacts were discarded. The red channel videos were used to categorize CSp or IT neurons.

#### Reaching decoding using RUSBoost.

Raw GCaMP6f traces (20 min) were z-scored, using the *zscore* function in MATLAB (Version R2024a, MathWorks Inc., RRID:SCR_001622) which computes as follows:

$$z_{i}=\frac{x_{i}\;-\;\mu }{\sigma }$$

Where $x_{i}$ is each data point of the Ca^2+^ trace, μ is the mean of the whole trace (20 min), and σ is the standard deviation of the trace. Then a binary RUSBoost ensemble classifier (*fitcensemble*, Method = “RUSBoost”) was trained for each mouse separately across the entire session. For each recorded cell, the signal (predictor) was partitioned using cross-validation (*cvpartition*), using 75% of the data for training and 25% for testing. The response variable for each mouse was a binary vector with ones at the timestamped peak of the reaches as well as at 3 frames before and after that peak, to cover the entire reaching period. To assess chance-level performance, additional control models were trained in which the z-scored Ca^2+^ signal of each cell was randomly shuffled (*randperm*, no repeats). Classifier performance was compared across groups using the F1 score, ensuring robustness to class imbalance.


$$F1=2\times \frac{\mathrm{Precision}\;\times \;\mathrm{Recall}}{\mathrm{Precision}\;+\;\mathrm{Recall}}$$

#### Ca^2+^ events detection.

Ca^2+^ signals were analyzed by behavioral states: reaching versus non-reaching epochs. We first identified non-reach epochs, defined as periods of at least 30 seconds during which the mouse did not perform any reaches. Once these periods were extracted, the remaining epochs were identified as reach epochs. Quantification of Ca^2+^ events from signal traces (e.g., frequency, AUC of individual Ca^2+^ transients) was performed on the IDEAS platform. First, the GCaMP6f traces of the cells identified by the CNMFe were denoised using the Deconvolution workflow on IDEAS, using the median method for estimating pixel noise, with a minimum noise frequency of 0.25 and a maximum noise frequency of 0.5. Then, the Ca^2+^ event detection algorithm was applied to the denoised traces. Briefly, baseline fluctuations were removed by subtracting the median trace using a 200 time bins sliding window and a 5-frame sliding average ^60^. Events were then extracted by searching for local maxima based on the following criteria: the amplitude at the maximum must be more than 2 standard deviations (SD) from the baseline, the width must be at least 10 frames when the mean intensity surrounding the maximum remains above 2 SD, and there must be a separation of at least 10 frames between two consecutive events ^60^. The event frequency and AUC were then extracted for the reach and non-reach epochs. To statistically compare the frequency and area under the curve (AUC) of Ca^2+^ events, outliers were identified using median absolute deviation method and removed prior to analysis. Frequency of all cortical pyramidal neurons was then analyzed using linear mixed-effects models (R’s *lmer()* from *lmerTest.*) with group, age, and their interaction as fixed effects, with mouse as the repeated measure random effect and an additional random intercept for each mouse-at-age combination (Fig. 2N–O). The significance of fixed effects was assessed using analysis of variance with Kenward-Roger’s degrees of freedom followed by Benjamini–Hochberg correction. To compare differences in Ca^2+^ events by cell subtypes (i.e., CSp versus IT neurons) under different behavioral states (i.e., reaching versus non-reaching), a linear mixed-effects model with fixed effects for cell subtype, age, group, their pairwise interactions, and their three-way interaction, with random intercepts for mouse and for age nested within mouse to account for repeated measurements was fit (Fig. 3C, D, G, H). Within each model, the Benjamini-Hochberg correction was applied across the age-specific group comparisons.

#### Correlation analysis.

Neural synchrony between cells was quantified using the Pearson correlation and evaluated separately within two behavioral conditions defined for each recording: the longest continuous period during which the mouse performed reaching tasks and the longest resting period without reaching behavior. The Pearson correlation coefficient was computed from pairs of extracted Ca^2+^ traces of the identified CSp or IT neurons. For each pair of cells, the correlation was calculated based on the raw fluorescence traces obtained from the calcium imaging recordings. We examined differences in neural synchrony between MitoPark and control mice while accounting for repeated measurements within animals. To evaluate group differences across the distribution of synchrony measures, we used smooth additive quantile mixed models applied to pooled cell coactivation data across observation time points (weeks) and animals. This modeling framework accounts for the non-independence of repeated measurements of coactivation within the same cells and mice. The model is specified as

$$Q_{\tau }(y|Length,\;Group,\;Week\;)=\beta_{0}+f(Length)+\beta_{1}\;Group\;+\beta_{2}\;Week\;+\beta_{3}\;Group\;\times \;Week\;+b_{Mouse\;}+b_{Mouse:Week\;}+b_{Cell1:Mouse:Week\;}+b_{Cell2:Mouse:Week\;}+\epsilon .$$

The $Q_{\tau }(y|Length,\;Group,\;Week)$ is the conditional τ-th quantile of the outcome variable. which was the cell coactivation measure, defined as the Pearson correlation between cells. The f(Length) is the smooth function of the length of the time window used to compute the coactivation measure, which adjusts for the effect of time window length. Week and mouse group (Mitopark vs. control) were included as categorical fixed effects, and their interaction was included to allow group differences to vary across weeks. To account for the hierarchical structure of the data, the model incorporated random effects for cells nested within week and mouse ) $b_{\mathrm{Cell}1:\mathrm{Mouse}:\mathrm{Week}}$ and $b_{\mathrm{Cell}2:\mathrm{Mouse}:\mathrm{Week}}$, week nested within mouse $b_{\mathrm{Mouse}:\mathrm{Week}}$, and mouse $b_{\mathrm{Mouse}}$.

Quantile regression models were fitted separately at quantile levels τ=0.1,0.25,0.5,0.75,0.9 to characterize different parts of the distribution of cell coactivation. For each quantile level τ and each week, we obtained the predicted τ-th quantile of cell coactivation for the Mitopark and control groups. Group differences were then derived from the fitted quantile regression models.

#### Peri-event Analysis.

Reach-related Ca^2+^ activity was performed using the peri-event Analysis workflow on the Inscopix IDEAS platform. This workflow quantifies changes in neural signals surrounding event timestamps, i.e., the endpoint of reaches, for each session. This is done at both the population level and for individual neurons, categorizing them as up-modulated, down-modulated, or non-modulated. To do so, we isolated bouts of reaches defined as those not preceded by another reach within at least a 2-second interval. Then the denoised Ca^2+^ traces and the timestamps of the first reach in a bout were fed to the peri-event analysis workflow, which outputs the 3 modulation categories, compared to a null distribution obtained from 1000 random shuffles of event time. The statistical window was set to ±1 sec around the onset of grasps, with a significance threshold of 0.05. For up- and down-modulated cells, we quantify the magnitude of modulation by measuring the difference in the ΔF/F values between the +1 and −1 sec time windows, and the variability by measuring the standard deviation across trials between −1 and 0 sec time windows. For group comparisons across ages (Fig. 4E–H), the up-modulated ΔF/F differences were log-transformed, and the down-modulated ΔF/F differences were transformed with −log(−ΔF/F difference). The standard deviations of neurons under both categories were log-transformed. Then, linear mixed-effects models for log-transformed data were fitted with fixed effects for group, age, and their interaction, and random intercepts for mouse and age, with age nested within mouse. The random intercept for mouse captures correlation between cells on the same mouse and the random intercept for age nested in mouse captures additional correlation from cells recorded in the same week. Within each model, the Benjamini-Hochberg correction was used for multiple comparisons.

#### Longitudinal registration.

The *CaImAn* Multi-Registration tool on the Inscopix IDEAS platform was used to track cells across sessions. This tool is built around the main function *register_multisession* from the registration algorithm implemented in *CaImAn* ^61^. The workflow was run across multiple sessions for each mouse, with x/y pixel shifts ranging from 10 to 50 pixels, 10 pixels maximum distance between matching centroids, and a maximum footprint disjunction of 0.7. The matched cells were used to compare cell modulation stability over time between groups, based on results from Peri-Event Analysis (matching cells across at least 3 sessions). To assess the stability of movement encoding by CSp neurons, agreement in categorical classifications across time was quantified using chance-corrected kappa statistics in a custom MATLAB script. For pairwise comparisons between consecutive timepoints, Cohen’s κ was computed as $\kappa =\frac{P_{o}-\;P_{e}}{1-\;P_{e}}$ where $P_{o}$ is the observed proportion of agreement and Pe is the expected agreement by chance. Overall agreement across all timepoints was assessed using Fleiss’ κ, defined as $K=\frac{\bar{P}-\bar{P_{e}}}{1-\bar{P_{e}}}$ where $\bar{P}$ is the mean observed agreement across subjects and ${\bar{P}}_{e}$ is the expected agreement based on category marginal proportions. Only cells with non-missing categorical assignments were included in the corresponding kappa computations.

#### Spectral clustering analysis.

Spectral clustering was performed using custom MATLAB scripts adapted from previously published methods ^47^. 2-sec segments of Ca^2+^ traces aligned to the onset of grasp (±1 sec time window) were extracted from individual cells and averaged across reaches. These mean segments were concatenated across control and MitoPark mice, and across cell types and age in a single matrix. Each segment was then z-scored using MATLAB's *zscore*() function to normalize activity across cells so that clustering was driven by the temporal dynamics of activity patterns (i.e., temporal shape of the response) rather than absolute response magnitude. Dimensionality reduction was performed using principal component analysis (PCA), and the first 6 principal components (PCs) were retained, based on the elbow of the explained variance curve. A k-nearest neighbor affinity matrix was then constructed in PCA space and used to compute the graph Laplacian for spectral embedding. Clustering was then performed using k-means, with the optimal number of clusters determined by the silhouette score. Cluster stability was assessed by subsampling and quantified using the adjusted Rand index. Cluster identities were finally mapped back to experimental group, cell subtype, and age for subsequent analysis. The proportion of cells in each cluster was also calculated, and Chi-square tests were used for group comparisons.

#### Binomial logistic regression.

Using custom MATLAB scripts, for each mouse, the denoised Ca^2+^ traces were z-scored using MATLAB’s *zscore()* function as described above. The traces were then aligned to timestamped grasp onset as previously described, and segments of 2 seconds around grasp onset were extracted as reach segments (see peri-event analysis for details). For comparison, randomly sampled non-overlapping segments of neural activity of identical duration (2 seconds) were drawn from periods without reaching, matched in number to reach segments (non-reach segments). Mice with recordings of fewer than 5 reaches at any age were excluded from this analysis. The extracted reach and non-reach segments were then concatenated across neurons and time to form reach-locked feature vectors. Reach versus non-reach segments were decoded using binomial logistic regression with 5-fold cross-validation. Within each training fold, dimensionality reduction was performed using PCA, retaining components that explained at least 85% of the variance. The classifier was fit on the training data using a logit link with Jeffreys-prior regularization, then applied to the held-out test data. Model performance was evaluated on held-out data using balanced accuracy and confusion matrices. Regression coefficients were back-projected from PCA space to the original neuron–time feature space and averaged across neurons within each cluster for each mouse for each week. This resulted in a time series for each mouse for each cluster for each week. Then separate generalized additive mixed models (GAMM) with restricted maximum likelihood for automatic smoothing were fit for each cluster within each week with a main effect of group, a smooth function of time, and a group-specific smooth interaction for MitoPark mice. Models additionally included a random intercept for mouse and autoregressive order-1 residual errors nested within mouse. This structure accounts for the correlation between observations from the same mouse that are close together in time. We then calculated the point-wise 95% confidence bands for the fitted smooth curves.

### Immunofluorescent staining and confocal imaging.

At the end of recordings, mice were anesthetized with Ketamine (100 mg/kg) + Xylazine (20 mg/kg) and transcardially perfused with 1X phosphate-buffered saline (PBS) for 5 min followed by 4% paraformaldehyde (PFA) for 5 min. The mouse head with the implant was then placed into 4% PFA at 4°C for 24 hours. After careful removal of the implants, the brain was extracted from the skull, then placed into 4% PFA overnight at 4°C. Brains were then rinsed three times using PBS and sectioned (70 μm) using a VT1000s vibratome (Leica Biosystems, Deer Park, IL; RRID:SCR_016495). Slices containing M1 and the striatum were collected. Brain sections containing the striatum were used for tyrosine hydroxylase (TH) immunohistofluorescence studies. Brain sections were rinsed three times using phosphate-buffered saline (PBS), and incubated with 2% normal donkey serum in 0.5% Triton-X-100 PBS solution for 1 hour, followed by incubation with primary antibody against TH [Mouse, 1:2000, cat#: MAB318, MilliporeSigma; RRID: AB_2201528)] overnight at room temperature. The brain sections were rinsed three times with PBS and incubated with the secondary antibody Alexa Fluor 647 donkey anti-mouse IgG (1:500, cat#715–605-150, Jackson ImmunoResearch Labs, RRID: AB_2340862) for two hours at room temperature. After three rinses with PBS, brain sections were mounted on slides (H-1200, Vector Laboratories) and coverslipped. Details of the immunofluorescent staining protocol can be found on Protocols.io: dx.doi.org/10.17504/protocols.io.b5s5q6g6. Immunofluorescence images were collected using a Nikon A1R Confocal Laser Scanning Microscope (RRID:SCR_020317). TH expression in the striatum and M1 was imaged with a 20x objective lens and quantified in ImageJ (NIH, https://imagej.net/, RRID: SCR_003070). TH immunofluorescence was quantified in the striatum and the cortex, and the striatal TH immunoreactivity was expressed as the ratio of striatum/cortex (Supplementary Fig. 1). This was done in 3 separate sections for each mouse and averaged. Details of confocal imaging and TH quantification can be found on Protocols.io: dx.doi.org/10.17504/protocols.io.3byl4jmxzlo5/v1 and dx.doi.org/10.17504/protocols.io.n2bvj85qngk5/v1.

To register the location of lens implants in brain atlas coordinates, brain slices with implantation tracks were imaged using either a slide scanner (ZEISS Axio Scan.Z1 Slide Scanner, RRID:SCR_020927) or a confocal microscope (Leica SP8 lightning confocal microscope, RRID:SCR_018169). Images were then processed using the EBRAINS service for image registration (QuickNII and VisuAlign, https://ebrains.eu/service/quicknii-and-visualign, RRID:SCR_022818). Real AP, ML and DV coordinates of the prism lens implant were thus identified and compared to target implantation coordinates.

### Statistics.

All data analyses were performed using the Inscopix IDEAS platform, GraphPad Prism 10 (Version 10, GraphPad Software, http://www.graphpad.com, RRID: SCR_002798), and custom scripts in MATLAB (MathWorks) and R (R Core Team). R packages lme4, emmeans, qgam, and mgcv were used. Figures were created using Adobe Illustrator (version 2024, https://www.adobe.com/products/illustrator.html, RRID:SCR_010279). Detailed statistics for each experiment can be found in the corresponding sections above, figure legends, and the raw data tables. Data were reported as mean and standard error of the mean (SEM). Statistically significant differences were identified at ** p < 0.05, ** p < 0.01, *** p < 0.001, **** p < 0.0001.

## Supplementary Material

Supplement 1

Supplement 2

## Data Availability

The raw and analyzed data generated throughout this study, as well as the Matlab and R codes used for data analysis, have been deposited and are publicly available from: 10.5281/zenodo.18295485.
