# Supplementary material for: Emergence of Task-Related Motor Cortical Dysfunction in Mice with Progressive Parkinsonism
Source: bioRxiv. 2026 Jun 2:2026.05.29.728524. Preprint. [Version 1] doi: 10.64898/2026.05.29.728524 (PMC13252005; doi:10.64898/2026.05.29.728524)
Supplement: Supplement 2 [file media-2.docx]

**
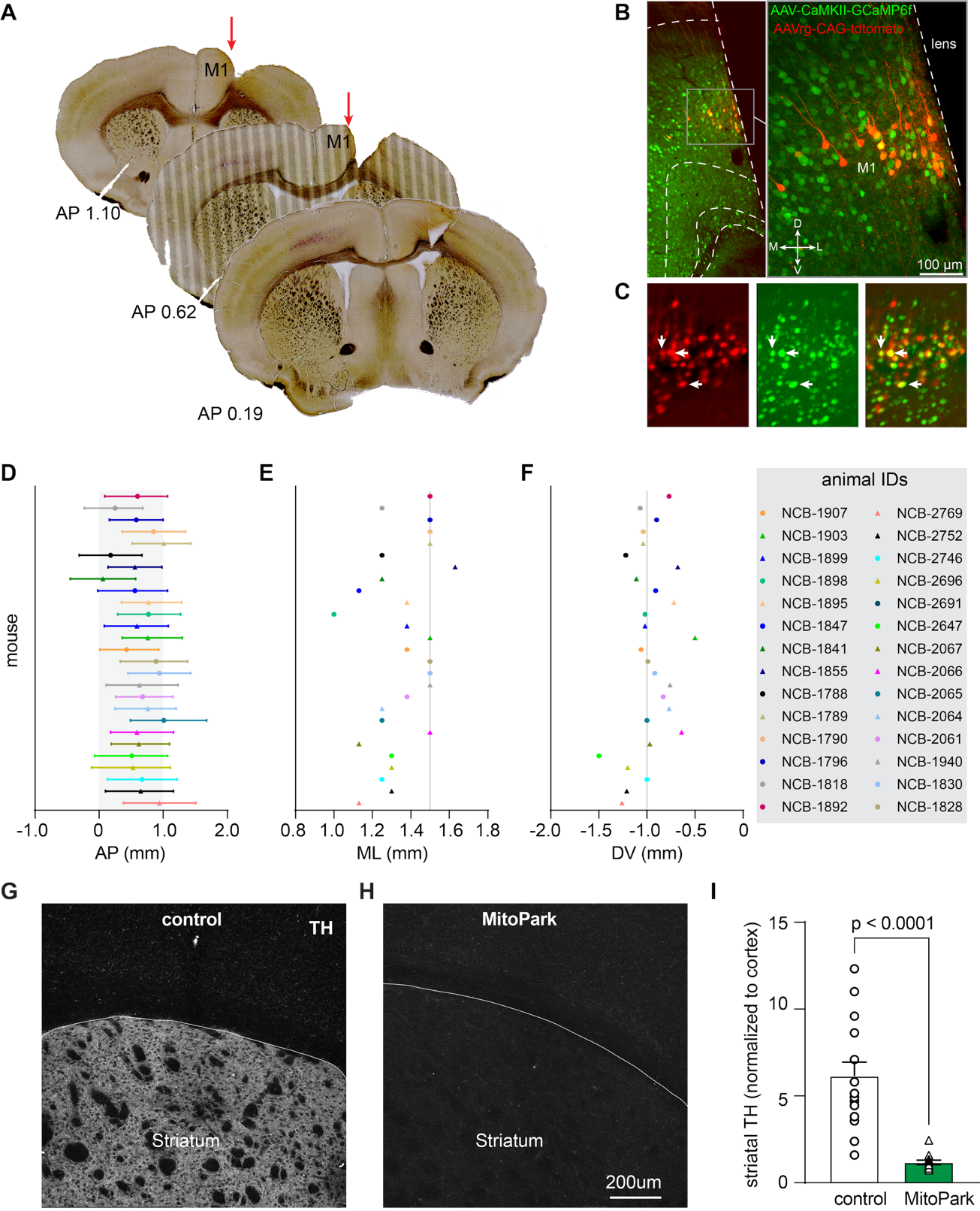
**

**Supplementary Figure 1. Histological validation of implantation site and degeneration of the nigrostriatal dopaminergic projection in MitoPark mice. A)** Representative images used for atlas registration. **B-C)** Representative *post hoc* confocal images (B) and *in vivo* field of view (FoV) of miniScope showing the laminar location of the center of FoV. Arrows in (C) highlight co-labeled CSp neurons in M1. **D-F)** The antero-posterior (D), medio-lateral (E), and dorso-ventral (F) coordinates of the GRIN lens implant of all mice used in this study. The gray shaded area in D and the gray vertical lines in E and F represent the target coordinates. Individual animals are listed on the right. **G-H)** Representative images of striatal TH staining in control and MitoPark mice. **I)** Striatal TH quantification. Control = 6.14 ± 0.8, N = 15 mice, MitoPark = 1.17 ± 0.13, N = 13 mice, p < 0.0001. Mann-Whitney U test.


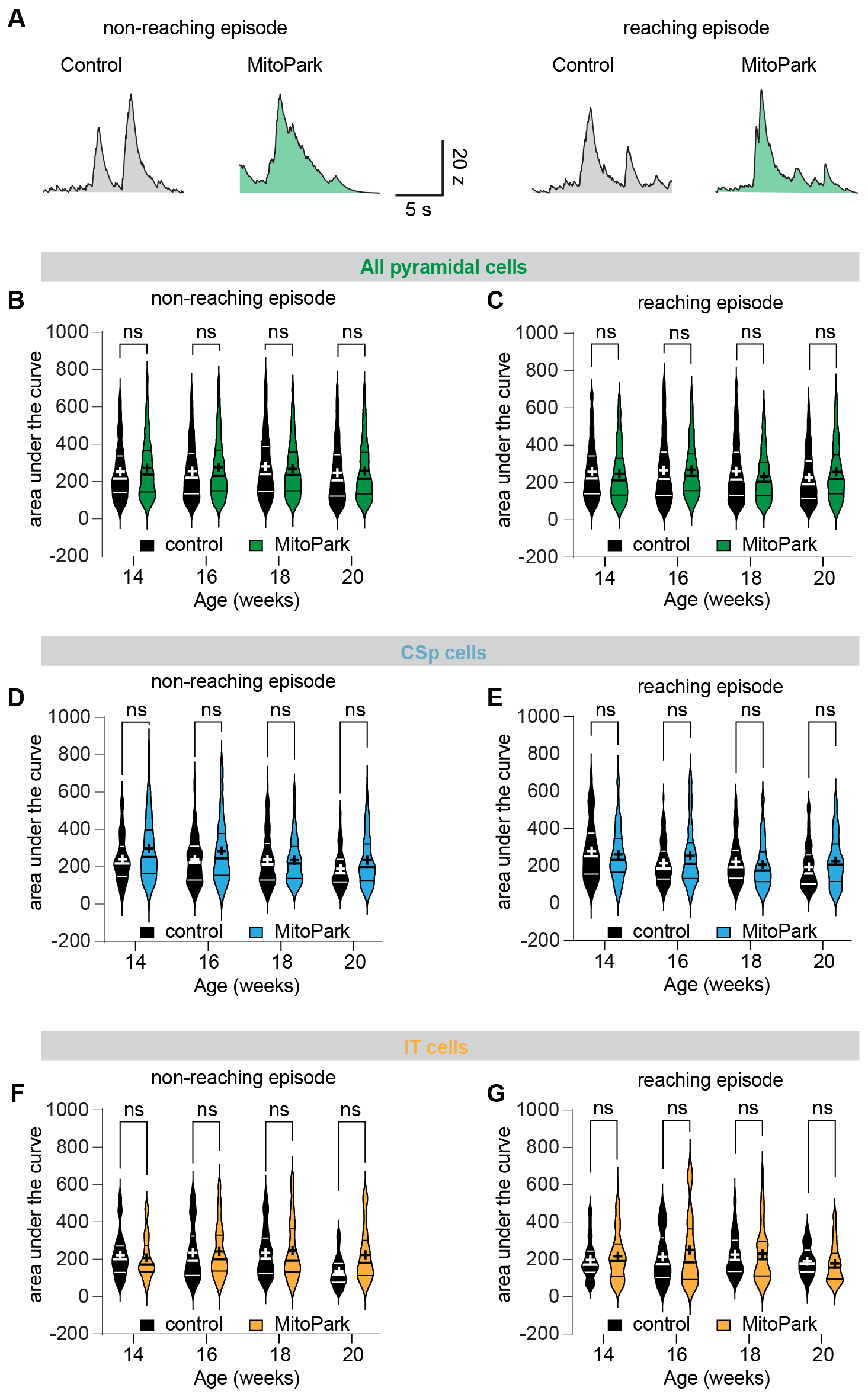


**Supplementary Figure 2. Area under the curve of Ca^2+^ traces of M1 pyramidal neurons. A)** Representative Ca^2+^ transients from a control mouse (gray) and a MitoPark mouse (green), during non-reaching epochs (left) and reaching epochs (right). **B-G)** Area under the curve of Ca^2+^ events averaged over non-reaching (B, D, F) and reaching epochs (C, E, G) of M1 pyramidal cells (B-C), CSp cells (D-E) and IT cells (F-G). Horizontal lines indicate median and IQR, cross (+) symbols indicate the mean. Linear mixed-effects model. Detailed numbers and statistics are available in the Source Data Table.

**
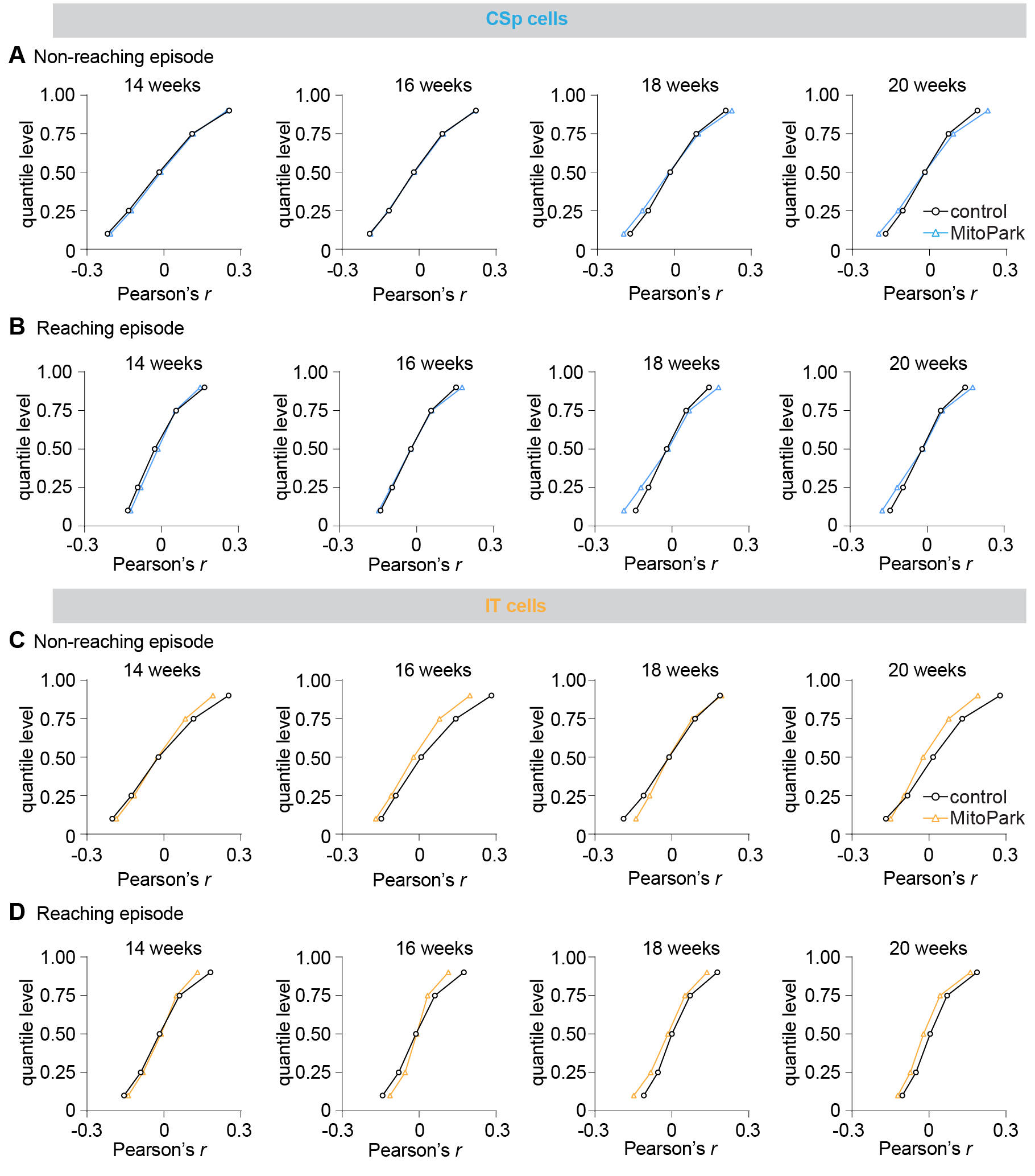
**

**Supplementary Figure 3. Correlation analysis of Ca^2+^ events in CSp and IT neurons.** Quantile regression models fitted at quantile levels τ=0.1,0.25,0.5,0.75,0.9 for Pearson’s *r* of CSp (A-B) and IT (C-D) cells during non-reaching (A-C) and reaching (B-D) epochs, showing no strong correlation of calcium signals in either group and cell subtypes. Detailed numbers and statistics are available in the Source Data Table.

**
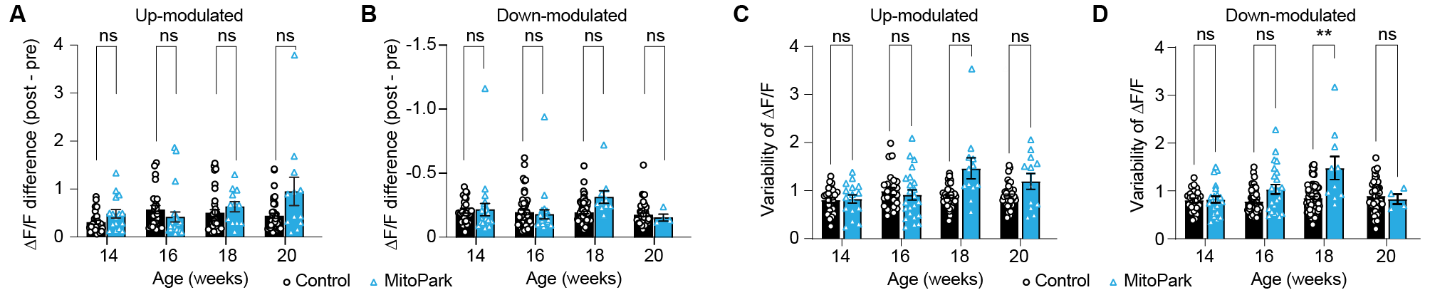
**

**Supplementary Figure 4. Movement modulation of CSp neurons in MitoPark mice and controls.** **A-B)** Summary results of the magnitude of modulation for up- (A) and down-modulated (B) CSp neurons. **C-D)** Summary results of the variability of dF/F (standard deviation across trials) averaged before grasp onset for up-modulated (C) and down-modulated (D) CSp cells. Linear mixed-effects model, followed by multiple comparisons with Benjamini-Hochberg correction. Detailed numbers and statistics are available in the Source Data Table.


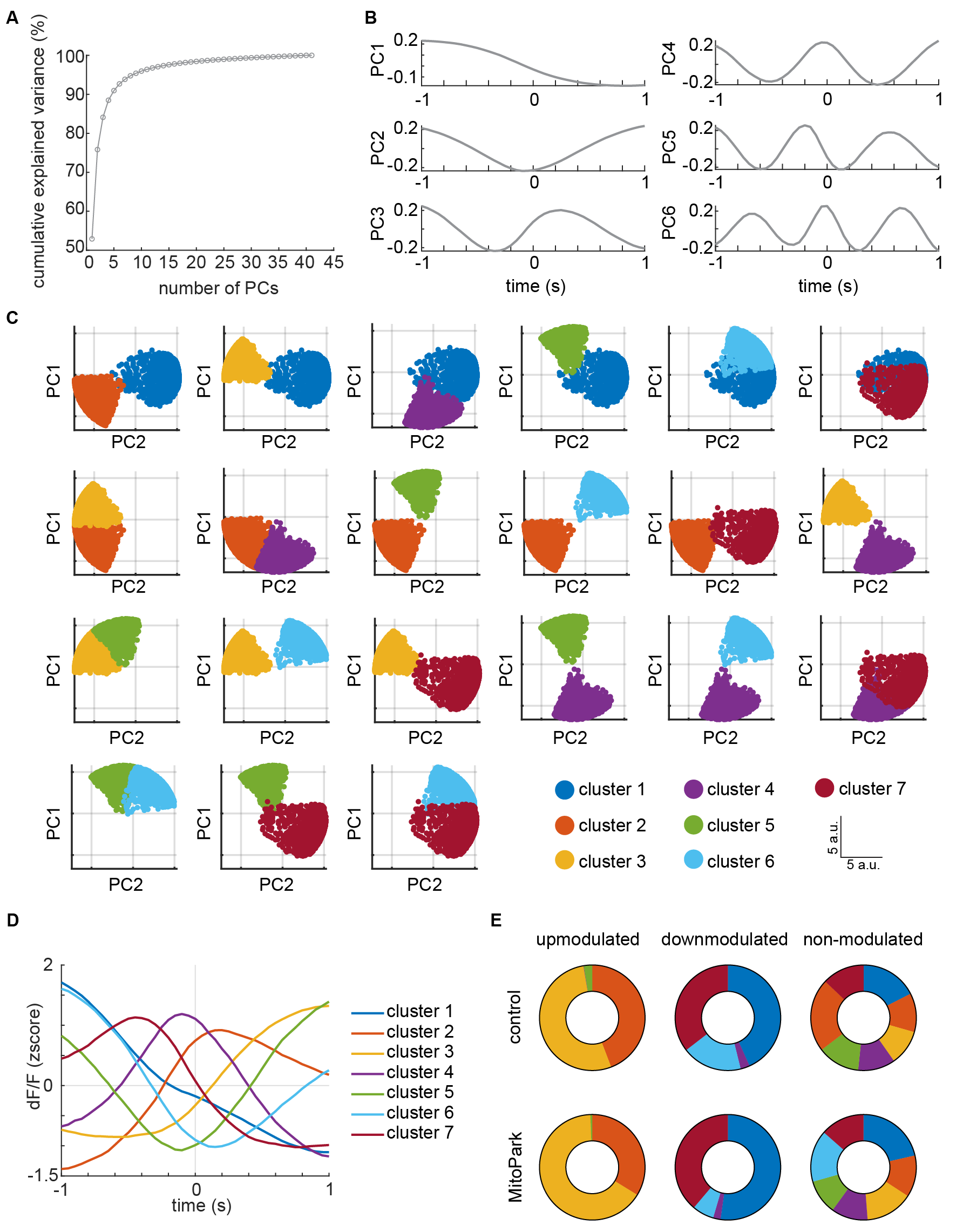


**Supplementary Figure 5. Spectral clustering analysis of M1 pyramidal neurons. A)** Cumulative explained variance plot. Elbow method was used, and 6 PCs were retained. **B)** Plots of the weights of the first 6 PCs. **C)** Cells projected onto 2D PC space to show clusters separation. Each subplot shows PC1 versus PC2 for one pair of clusters. **D)** Mean traces of each cluster showing the different functional temporal profiles identified, averaged across age and experimental groups for representative purposes. **E)** Overlap between neurons identified as upmodulated (left), downmodulated (middle), and non-modulated (right) and cluster identity in control (top) and MitoPark mice (bottom), pooled across ages. Colors denote cluster identity and are consistent across panels.

**
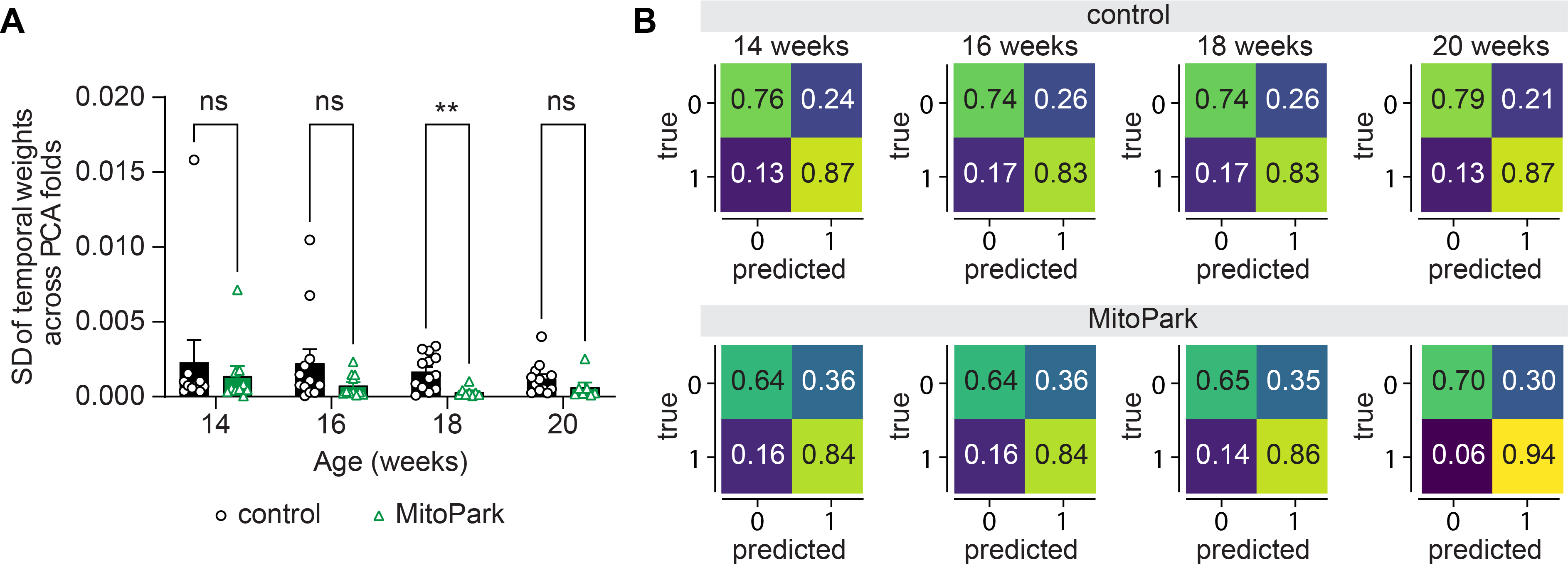
**

**Supplementary Figure 6. Binomial logistic regression on M1 pyramidal cells quality control parameters. A)** SD of absolute temporal weights across PCA folds, for each mouse, showing minimal variability in both groups, and no statistically significant differences between them. Linear mixed effect model followed by Benjamini-Hochberg correction. **B)** Confusion matrices averaged across PCA folds for control (top) and MitoPark mice (bottom) groups, reported as fractions. Detailed numbers and statistics are available in the Source Data Table


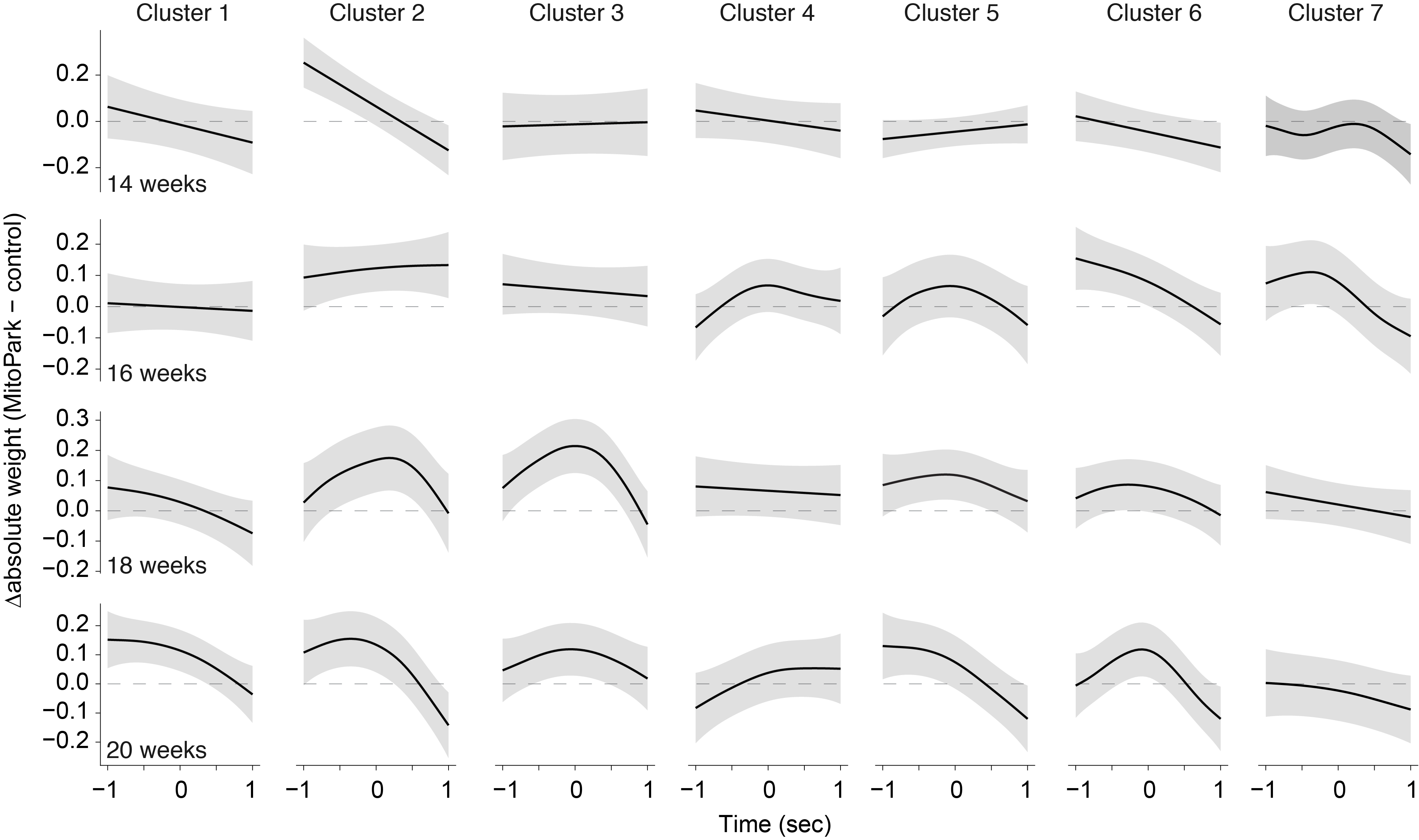


**Supplementary Figure 7. Difference between the reach encoding curves in control and MitoPark mice.** For each week and each cluster, a GAMM with autocorrelated errors was fit to predict the absolute temporal weights from time, with separate smooths fit for MitoPark and control. The difference curves were obtained by subtracting the smooth for control from MitoPark. Gray bands indicate point-wise 95% confidence bands.
